# Supplementary material for: Chromothripsis during telomere crisis is independent of NHEJ, and consistent with a replicative origin
Source: Genome Res. 2019 May;29(5):737–49. doi: 10.1101/gr.240705.118 (PMC6499312; doi:10.1101/gr.240705.118)
Supplement: Supplemental Material [file supp_gr.240705.118_Supplemental_file_1.zip › contigs/annotated_contigs/DB73/contig.2.DB73_length_389_mean_cov_5.01285347044.docx]

**DB73_length_389_mean_cov_5.01285347044**

```
CCAGGCATCAGGAGCAAGCACTTCTGAGCCTGCTAGGGCAGGGGGGCCTTCCTGGACCGCCAAGAGTTCAAGGATGCCTGAGTCTGCAG
 >chr3:191691428-191691525 + E=7e-47
CTATGGTT|AAATAAAATTTAAATAA|ACCCAAATCAATCGAGGACAATTTTTTTTTTCTTCTTTGGAAGAGCTTAGACATACTTATCA
                          >chr3:190043093-190043368 + E=3e-154
TGTGTTTCTTGCTCCAGGCAGTGGCAAGATATTTTACTCTGTGACTGTCTCTTCTTAATATCTGTTGCAGCCCACTGAGGTTTTATACC

CTGGGGATTTAGAATCATGTGGCAAAAAGTTGTTGCTGTTGAGGAGGAAGAGATGTGTAAAAACAGCAAATAGCGTACTGGTTTATTCC

ATGTGCTTGTGCGACTGATGCTTGGGCTTAGCCAT
```
